# Gene Co-expression Networks Identifies Common Hub Genes Between Cutaneous Sarcoidosis and Discoid Lupus Erythematosus

**DOI:** 10.3389/fmed.2020.606461

**Published:** 2020-11-25

**Authors:** Melissa A. Nickles, Kai Huang, Yi-Shin Chang, Maria M. Tsoukas, Nadera J. Sweiss, David L. Perkins, Patricia W. Finn

**Affiliations:** ^1^Department of Medicine, University of Illinois at Chicago, Chicago, IL, United States; ^2^Department of Dermatology, University of Illinois at Chicago, Chicago, IL, United States; ^3^Division of Rheumatology, University of Illinois at Chicago, Chicago, IL, United States

**Keywords:** WGCNA, co-expression network, cutaneous sarcoidosis, discoid lupus erythematosus, hub genes

## Abstract

In this study we analyzed gene co-expression networks of three immune-related skin diseases: cutaneous sarcoidosis (CS), discoid lupus erythematosus (DLE), and psoriasis. We propose that investigation of gene co-expression networks may provide insights into underlying disease mechanisms. Microarray expression data from two cohorts of patients with CS, DLE, or psoriasis skin lesions were analyzed. We applied weighted gene correlation network analysis (WGCNA) to construct gene-gene similarity networks and cluster genes into modules based on similar expression profiles. A module of interest that was preserved between datasets and corresponded with case/control status was identified. This module was related to immune activation, specifically leukocyte activation, and was significantly increased in both CS lesions and DLE lesions compared to their respective controls. Protein-protein interaction (PPI) networks constructed for this module revealed seven common hub genes between CS lesions and DLE lesions: TLR1, ITGAL, TNFRSF1B, CD86, SPI1, BTK, and IL10RA. Common hub genes were highly upregulated in CS lesions and DLE lesions compared to their respective controls in a differential expression analysis. Our results indicate common gene expression patterns in the immune processes of CS and DLE, which may have indications for future therapeutic targets and serve as Th1-mediated disease biomarkers. Additionally, we identified hub genes unique to CS and DLE, which can help differentiate these diseases from one another and may serve as unique therapeutic targets and biomarkers. Notably, we find common gene expression patterns in the immune processes of CS and DLE through utilization of WGCNA.

## Introduction

Cutaneous sarcoidosis (CS), discoid lupus erythematosus (DLE), and psoriasis are immune-related cutaneous disorders with different pathologies and clinical presentations. CS occurs in up to one third of patients with systemic sarcoidosis, an inflammatory disease characterized by non-caseating granulomas (1). CS is often considered the “great imitator” in dermatology due to its large range of morphologies, including papules, plaques, lupus pernio, and scar psoriaform and ulcerative lesions (1). DLE is the most prevalent type of chronic cutaneous lupus erythematosus characterized by pathogenic autoantibodies and immune complexes (2). The most common presentation of DLE is coin-shaped plaques on the scalp and ears (2, 3). DLE can occur as a skin manifestation of systemic lupus erythematosus (SLE) in up to 20% of patients (2, 3). Psoriasis is a common skin condition that affects over 7 million Americans (4). The hallmark of immune dysfunction in psoriasis is uncontrolled keratocyte proliferation and differentiation (5). Plaque psoriasis is the most common subtype, presenting with well-defined areas of erythematous plaques with silvery scales (5). The severity of psoriasis can greatly vary, with the joints being affected in 20–30% of patients (6). While psoriasis was traditionally considered a Th1-mediated disease, recent studies suggests that psoriasis may be predominantly Th17-mediated (7). In contrast, both CS and DLE may be predominantly Th1-mediated diseases (8, 9).

Within the past 20 years, biological treatments for several skin diseases, including psoriasis, atopic dermatitis, urticaria, and pemphigus vulgaris have emerged as major therapeutic breakthroughs (10). First-line therapy for cutaneous sarcoidosis consists of corticosteroids and second-line therapies consist of tetracyclines, hydroxychloroquine, and methotrexate. Biologics, e.g., anti-TNF, have been used to treat chronic or resistant cutaneous sarcoidosis with improvement or worsening of disease (11). A limited number of studies support the use of both infliximab and adalimumab as third-line therapies for cutaneous sarcoidosis, with some reports of etanercept, rituximab, golimumab, and ustekinumab being successful as well (12). For DLE, first line therapies include photoprotection in conjunction with topical or oral corticosteroids, topical calcineurin inhibitors, and systemic antimalarial therapy (2, 3, 13). Refractory lesions may be treated with intralesional corticosteroid injections (2, 13). Chronic DLE lesions that are not responsive to topical corticosteroids or topical calcineurin inhibitors may be responsive to intralesional corticosteroid injections (2). Intravenous immunoglobin, rituximab, and dapsone have successfully treated cutaneous lupus lesions in a limited number of studies, as well as toxilizumab and anti-CD4 antibody in single reports (14). For mild to moderate psoriasis, first-line therapies include topical therapies including corticosteroids, vitamin D3 analogs, and combination products (15). Moderate to severe psoriasis can be treated with systemic therapies such as phototherapy, acitretin, methotrexate, and cyclosporine (15). For patients who do not respond to systemic therapies, biologics may be used. Infliximab has been found to be the most effective, followed by ustekinumab, adalimumab, and etanercept (15). These therapies do not address the presence of concomitant diseases of sarcoidosis and psoriasis. For example, anti-TNF in sarcoidosis may induce psoriasis skin lesions. Thus, we undertook this study to dissect the common and differentially expressed pathways among these three diseases.

Biological therapies must target a specific immune component that plays a key role in disease pathogenesis. Ideally, treatment should be directed to a patient-specific target (16). We have previously used gene co-expression networks to identify genes and molecular pathways of a disease state associated with clinical traits (17), as well as identifying similar immunological mechanisms between sarcoidosis and idiopathic pulmonary (18). In this study, we characterized commonly altered biological pathways in cutaneous sarcoidosis (CS), discoid lupus erythematosus (DLE), and psoriasis using gene coexpression networks. We created gene co-expression networks of microarray data from two previous studies. The first study found that active CS skin lesions showed several thousand differentially expression genes compared to non-lesional skin in CS patients and healthy controls. These differentially expressed genes showed a strong Th1 profile of sarcoidosis and expression of interleukin (IL)-23 and IL-23R with limited expression of other Th17 pathway genes (8). The second study found that DLE skin lesions demonstrated a predominance of IFN-γ-producing Th1 cells and an absence of IL-17-producing Th17 cells compared to psoriasis skin lesions (9).

In this study, we hypothesized that there would be common gene expression patterns between CS and DLE due to the similarities between the two diseases. Both CS and DLE are related to systemic disease, have a greater prevalence in African American populations (2, 19), and are predominantly Th1-mediated (8, 9). To investigate this hypothesis, microarray expression data with weighted gene co-network analysis (WGCNA) was applied. Since genes with similar expression patterns are likely to be functionally related, WGCNA clusters genes with correlated expression profiles into groups known as modules. WGCNA was used to identify the most relevant module in immune-related skin disorders. Hub genes within the module were identified using intramodular connectivity and protein-protein interaction (PPI) networks. The hub genes were further characterized by differential gene expression (DGE) analysis. We propose that characterization of commonly altered biologic pathways in CS, DLE, and psoriasis may uncover immunological targets and/or biomarkers.

## Methods

### Data Collection and Preprocessing

Microarray data and associated clinical data was obtained from the NCBI Gene Expression Omnibus (GEO). Dataset 1 (GSE32887) (8) included 15 skin samples from CS lesions, 11 skin samples of non-lesional skin (NLS) on the same patients with CS, and 5 skin samples from healthy volunteers (control 1). Dataset 2 (GSE52471) (9) included 7 skin samples from DLE lesions, 18 skin samples from psoriasis lesions, and 13 skin samples from healthy volunteers (control 2). Both datasets were generated using Affymetrix Human Genome U133A 2.0 Array. The *collapseRows()* function was used to filter the probes to include only unique genes that were present in both datasets.

### Co-expression Network Construction

The WGCNA package on R (20) was used to construct co-expression networks of both datasets. We utilized code from (21). Since both datasets used the same platform, they were comparable. To create a scale-free network, an appropriate soft power was selected to promote strong connections between genes and filter out weak ones. Pearson correlation was used to measure the concordance of pair-wise genes. The Pearson correlation matrix was transformed into a weighted network for each dataset using the *adjacency()* function. Dataset 1 was used to construct modules. The dynamic tree-cutting function with a cut height of 0.99, a minimum cluster size of 30, and deep split of 3 identified modules with similar expression patterns. Modules are given arbitrary color names for easier tracking.

### Identification of Module of Interest and GO Enrichment Analysis

The modules constructed from dataset 1 were mapped onto dataset 2. A preservation Z-score summary for each module was calculated using the *modulePreservation()* function in order to identify highly preserved modules between the two datasets. A Z-score of greater than five was used as the threshold for module preservation (21). The*GOenrichmentAnalysis()* function in WGCNA was used to annotate each module with significant biological functions. Module eigengenes (ME), which can be interpreted as the level of expression of each module within each sample, were obtained for all samples across studies. We performed a 3-group comparison using a single-factor ANOVA to compare MEs of preserved modules between the CS lesions, NLS, and controls in dataset 1. We repeated this procedure for DLE, psoriasis, and controls for dataset 2. For modules with significant ANOVA results, we performed follow-up pairwise *t*-tests assuming unequal variance between each subgroup in each dataset. The module which demonstrated a significant difference between case/control status in both datasets was investigated further.

### Identification of Hub Genes in Module of Interest

The genes in the module of interest were ranked by intramodular connectivity. The top 5% of genes in dataset 1 and dataset 2 were considered potential hub genes (22). The lists were cross-referenced to identify overlapping genes. We also used a second method to identify hub genes. The 2,000 genes (the maximum allowed) with the highest intramodular connectivity for each dataset were entered into the STRING (23) database to construct a protein-protein interaction (PPI) network. The minimum protein interaction score was set to high confidence (0.7). The output from the PPI network was imported into Cytoscape (24), an open source software platform for visualizing complex networks. The top 5% of genes with the highest degree were considered potential hub genes and the lists from the two datasets were cross referenced to identify overlapping genes (25). We termed genes that were common to both network types and common between datasets as “hub genes.” Gene expression of hub genes was compared between disease groups using Kruskal-Wallis test and *post-hoc* Dunn's test with Bonferroni correction for each gene.

### Validation of Hub Genes Using Differential Gene Expression (DGE) Analysis

The limma package (26) on R was used to identify differentially expressed genes between CS lesions and DLE lesions vs. their respective controls to validate our results. No covariates were used in the limma model because case/control status was the only variable. The *lmFit()* function and empirical Bayes method in the limma package was used to analyze the genes. The *topTable()* function summarized the results from the linear fit. We used the criteria of *p* < 0.01 and log2 (fold-change) to define differentially expressed genes. We adjusted our *p*-values via the Benjamini-Hochberg Procedure.

## Results

### Co-expression Network Construction Identifies a Module of Interest

Our filtering process resulted in 12,991 genes to use in network construction. The co-expression network resulted in 14 modules, six of which were preserved between datasets. Of the six preserved modules, two modules were related to cellular division, one was related to biosynthesis, one was related to the nucleus, one was related to sensory perception, and one was related to leukocyte activation. The top 10 GO terms for these preserved modules can be found in File 1. All *P*-values reported have been adjusted for multiple comparisons. While all six preserved modules in dataset 2 revealed significant differences between case/control status (*p* < 0.01), only the leukocyte activation module significantly differed between case/control status in dataset 1 (*p* < 0.001). We therefore selected the leukocyte activation module as our module of interest. The module of interest was significantly increased in both the CS lesions and DLE lesions compared to their respective controls (*p* < 0.001). There was no significant difference between NLS and control in dataset 1. Psoriasis showed decreased expression of the leukocyte activation module compared to both DLE (*p* < 0.001) and controls in dataset 2 (*p* < 0.05).

### Hub Genes Involved in Sarcoidosis and Lupus Pathogenesis Are Identified

**Figure 1:**
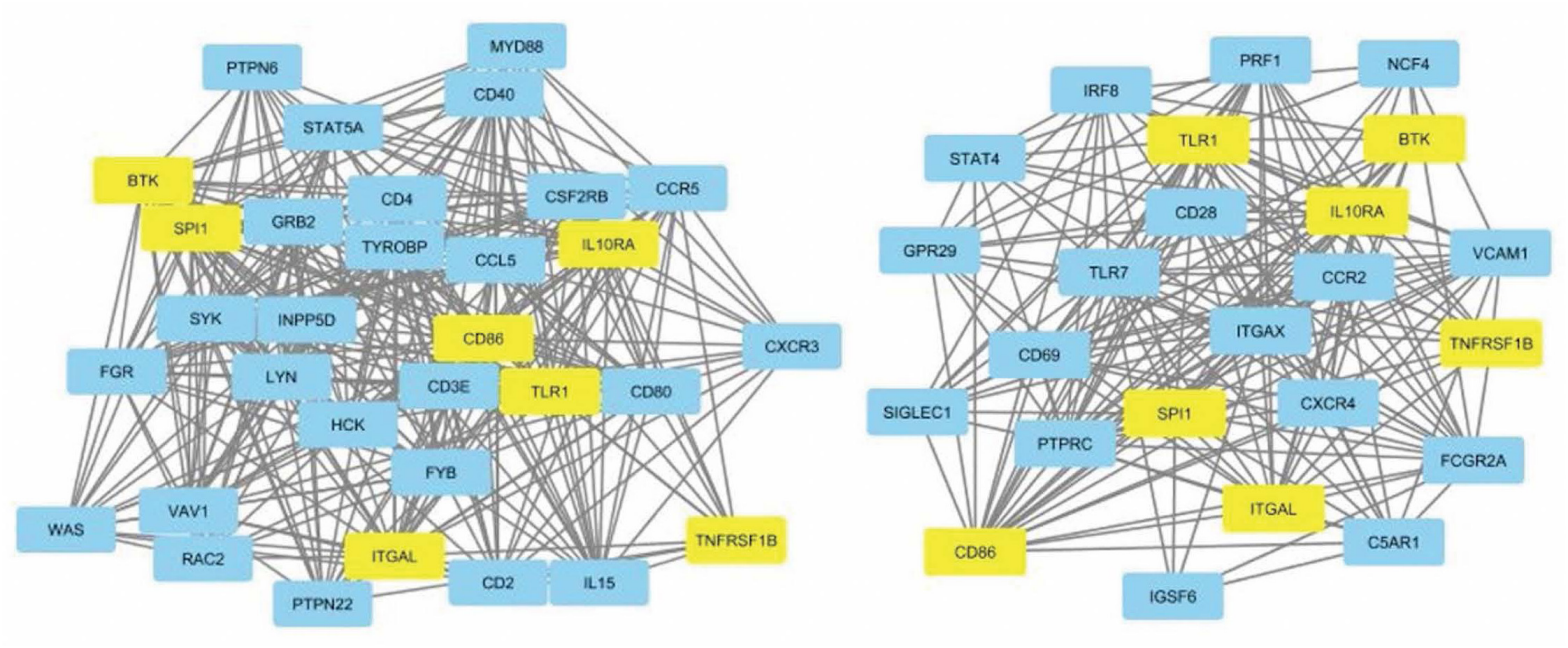
Protein-protein interaction network for dataset 1 showing nodes with 20+ connections (left). Protein-protein interaction network for dataset 2 showing nodes with 10+ connections (right). Common hub genes are highlighted in yellow. The network is derived from the leukocyte activation module.

**Figure 2:**
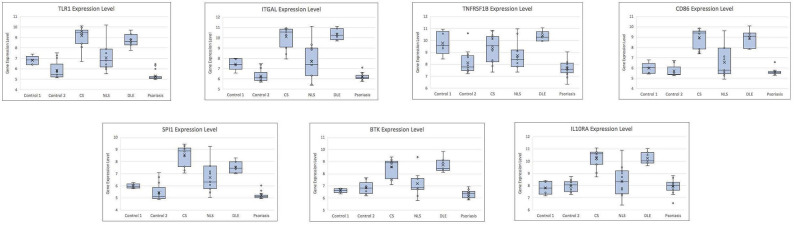
Box plots showing gene expression level for each hub gene by case/control status. CS, cutaneous sarcoidosis; NLS, non-lesional skin of patients with cutaneous sarcoidosis; DLE, discoid lupus erythematosus.

The leukocyte activation module contained a total of 3,511 genes. From the co-expression network, the two datasets had 21 potential hub genes in common. From the PPI network, the two datasets had 74 potential hub genes in common. We found seven genes that were hubs in both network construction methods: TLR1, ITGAL, TNFRSF1B, CD86, SPI1, BTK, and IL10RA (Figure 1). All seven hub genes were upregulated in both the CS and DLE compared to their respective controls (*p* < 0.05). Additionally, all seven genes were significantly upregulated in the CS lesions compared to NLS, which did not show any significant differences from their controls. The psoriasis lesion samples showed decreased expression of BTK and TNFRSF1B compared to controls (Table 1). The seven hub genes were unique to the leukocyte activation module and were not found in any other preserved module. The expression level of each hub gene based on case/control status is demonstrated in Figure 2.

**Table 1:** Adjusted *P*-values demonstrate significant differences in hub gene expression levels between groups in each dataset.

**Gene name**	**Adjusted** ***P*****-values of hub gene expression values**					
	**Dataset 1**			**Dataset 2**		
	**CS vs. control**	**NLS vs. control**	**CS vs. NLS**	**DLE vs. control**	**Psoriasis vs. control**	**DLE vs. psoriasis**
BTK	0.008^*^	NS	0.0115^*^	0.0129^*^	0.0357^**^	< 0.0001^*^
CD86	0.0024^*^	NS	0.0015^*^	0.0001^*^	NS	0.0005^*^
ITGAL	0.0057^*^	NS	0.0015^*^	0.0002^*^	NS	0.0004^*^
IL10RA	0.0011^*^	NS	0.0023^*^	0.0004^*^	NS	0.0002^*^
SPI1	0.0008^*^	NS	0.0035^*^	0.0004^*^	NS	0.0003^*^
TLR1	0.0071^*^	NS	0.0011^*^	0.0153^*^	NS	< 0.0001^*^
TNFRSF1B	0.0017^*^	NS	0.0027^*^	0.0007^*^	0.0252^**^	0.0001^*^

* *Increased expression level*;

** *Decreased expression level*.

*CS, cutaneous sarcoidosis; NLS, non-lesional sarcoidosis lesion; DLE, discoid lupus erythematosus*.

Seventeen hub genes were found only in dataset 1, including CD40, CCR5, CCL5, MYD88, IL15, and CXCR3. Six hub genes were found only in the dataset 2, of note TLR7, FCGR2A, and CCR2. The listed genes have primarily been indicated in the pathogenesis of sarcoidosis or lupus in the literature (27–30). Full gene lists can be found in Table 2.

**Table 2:** Hub genes that are unique to disease-type identified by intramodular connectivity and protein-protein interaction (PPI) networks.

**Cutaneous sarcoidosis hub genes**	SYK, CD40^*^, CD80^*^, CCR5^*^, CCL5, MYD88, IL15, LYN, RAC2, GRB2^*^, STAT5A^*^, VAV1, CXCR3^*^, PTPN6, CD33^*^, HCK, FGR
**Discoid lupus erythematosus hub genes**	**PTPRC, TLR7, ITGAX, FCGR2A, CCR2, PRF1**

* *Indicates that the gene was unique to that disease-type in the differential gene expression analysis*.

### Common Hub Genes Are Highly Upregulated in Differential Expression Analysis

**Figure 3:**
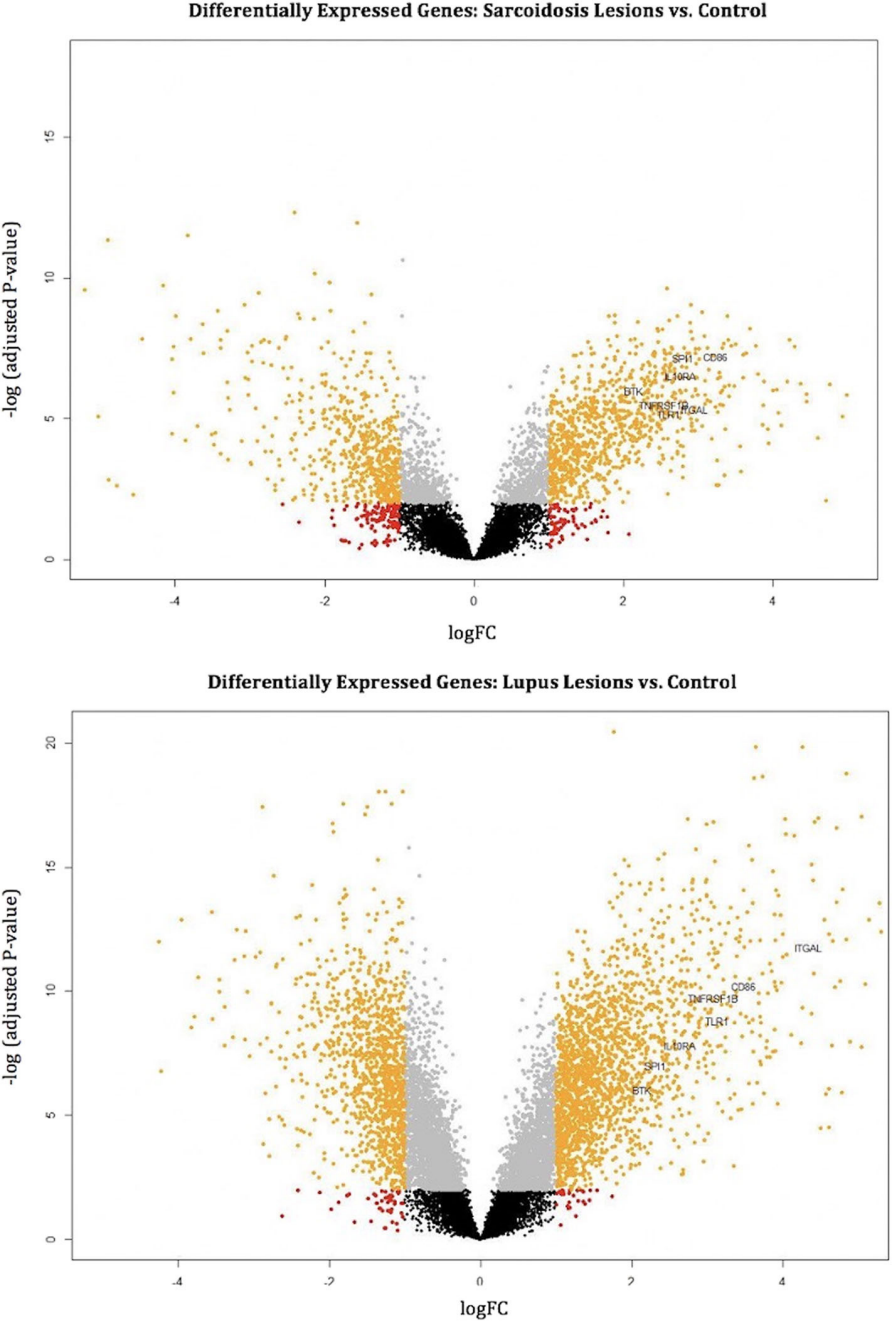
Volcano plots mark differentially expressed genes (orange dots) in cutaneous sarcoidosis lesions vs. control (top) and discoid lupus erythematosus lesions vs. control (bottom). The orange dots represent differentially expressed genes using the criteria of *p* < 0.01 and log2 (fold-change). Black, gray, and red dots are genes that do not meet the criteria of a differentially expressed gene. The common hub genes (TLR1, ITGAL, TNFRSF1B, CD86, SPI1, BTK, and IL10RA) are marked.

The common hub genes were found to be individually upregulated in disease states compared to controls. The DGE analysis demonstrates that as a group, the common hub genes are highly upregulated compared to other DEGs in the datasets for both CS and DLE vs. respective controls. Volcano plots of DEGs with labeled hub genes are shown in Figure 3.

All 17 hub genes unique to dataset 1 showed significant upregulation in the DGE analysis of CS lesions vs. control. Ten of the 17 hub genes unique to dataset 1 were also significantly upregulated DEGs in the DLE vs. control analysis. The remaining seven were upregulated DEGs only in the CS vs. control analysis (Table 2). Of the six hub genes significant only to dataset 2, all six were upregulated in DLE lesions vs. control, as well as the CS vs. control analysis. Thus, in the DGE analysis there were only seven hub genes that were unique to CS and no hub genes that were unique to DLE.

## Discussion

Our findings suggest that there may be common gene expression patterns in the immune processes of CS and DLE. Since there were no significant differences between control and NLS, it appears that the gene dysregulation is confined to the sarcoidosis lesions. Additionally, psoriasis does not demonstrate the same patterns of gene expression differences as demonstrated in CS and DLE, suggesting that similarities between CS and DLE may extend beyond being immune-related skin disorders. The common hub genes we identified may be further investigated as biomarkers of Th1-driven inflammatory disorders. These genes may represent underlying drivers of Th1-skewed immune disease and could serve as a therapeutic target in CS and DLE. We also identified hub genes that are unique to CS and DLE, which can help differentiate these diseases from one another and may serve as unique markers rather than general Th1-mediated disease markers.

Closer examination of the seven identified hub genes reveals involvement of pathways that activate the immune system. Some of these genes encode for proteins that are already therapeutic targets. For example, TNFRSF1B encodes a protein that is a member of the TNF-receptor superfamily, which mediates the recruitment of anti-apoptotic proteins (31, 32). TNF-α inhibitors are currently used to treat a variety of immune-related disorders including sarcoidosis, lupus, and plaque psoriasis (33). However, TNF-α inhibitors do not consistently work for sarcoidosis (34) and are also complicated in SLE treatment due to the fact that TNF-α inhibitors can induce lupus (35). Future studies must utilize gene-regulatory networks to stratify phenotypes of sarcoidosis and lupus that are responsive to specific therapies.

Other hub genes identified encode for immune system related proteins that have shown promise as biomarkers or targets. CD86 encodes a protein that is expressed on antigen presenting cells (APCs) and serves as the costimulatory signal for T-cell activation (31). In sarcoidosis patients, alveolar macrophages (AM) act as APCs and express high levels of CD86 (31) to stimulate T-cell activation. BTK, which plays a crucial role in B-cell development, has been identified as having multiple roles in the production of autoantibodies and the pathogenesis of lupus (36). BTK inhibitors serve as a promising new therapeutic target and are currently being tested to treat lupus in animal models (37). TLR1 is a member of the toll-like receptor (TLR) family, which plays a fundamental role in pathogen recognition and innate immunity. Toll-like receptors recognize pathogen-associated molecular pathways (PAMPS) that are expressed on infectious agents (38). TLR1 in particular is expressed at higher levels than other TLRs and has been associated with infectious diseases that affect the skin, including Leprosy (39) and Lyme Disease (40). TLRs are thought to play a role in both sarcoidosis and lupus pathogenesis (41, 42), and has been suggested as a potential therapeutic target for lupus (43).

Hub genes may be useful as biomarkers for disease activity as well. IL10RA is a receptor for interleukin 10, an anti-inflammatory cytokine. IL-10 is increased in active pulmonary sarcoidosis as a compensatory response to increased expression of proinflammatory cytokines (44). IL-10 mediates disease activity of SLE (45). Together, the genes identified are those known to influence inflammatory responses.

The hub genes unique to CS and DLE are also potentially useful as disease markers and treatment targets. Of the 17 hub genes unique to the sarcoidosis samples, many are involved in T-cell activation and proliferation, such as CD40, CD80, CCR5, CCL5, and IL15, or cellular signaling, such as SYK, MYD88, and CXCR3. Some of the hub genes have been indicated in the pathogenesis of sarcoidosis. For example, CD40 which is required to activate APCs, is expressed in higher levels on AMs in patients with sarcoidosis (46). The expression of CD40 correlates with CD86, a common hub gene (46).

The C-C chemokine receptor 5 (CCR5) binds to chemokine ligand RANTES (CCL5) and is expressed on T-cells and macrophages. CCR5 mRNA is increased in bronchoalveolar lavage fluid (BALF) of sarcoidosis patients and may serve as a marker of pulmonary disease (47). Furthermore, certain CCR5 haplotypes are associated with sarcoidosis. The CCR5 haplotype HHC is strongly correlated with parenchymal lung disease in sarcoidosis, however, appears not to increase susceptibility to sarcoidosis and is only relevant after disease induction (48).

As a whole, we identified sarcoidosis genes that reflect immune cell activation. In contrast, the unique genes of the lupus group are more involved in pathogen recognition and degradation, such as TLR7, and FCGR2A. Some FCGR2A polymorphisms may increase susceptibility and development of SLE in certain ethnic populations (30). Data from mouse models have shown that TLR7, which is involved with PAMP recognition, serves a pathogenic role in the development of SLE, while TLR9 serves a protective role (28). Altered expression of TLR7 and TLR9 has been suggested as a biomarker to identify a subset of SLE patients that may respond to a targeted therapeutic approach (29). CCR2+ T-cells, which aid in monocyte chemotaxis, are found to be selectively decreased during SLE flares and could potentially serve as a biomarker (27).

Together, our study indicates that our methodology may be informative in proposing key gene regulatory points in the immune processes of sarcoidosis and lupus. Notably, we find common gene expression patterns in the immune processes of CS and DLE. We have utilized WGCNA to identify genes that may underlie the pathogenesis of Th1-mediated skin disorders. The study limitations include lack of clinical data regarding therapy or systemic involvement, as well as sample collection at a single time point. Future analysis of the skin lesions at multiple points with functional interruption would be necessary to characterize the specific roles of hub genes in disease pathogenesis. We underscore that our demonstration of high intramolecular connectivity of the hub genes strongly suggests regulatory roles. We have identified hub genes that have been previously identified, as well as novel gene candidates for lupus and sarcoidosis. Lupus is a systemic autoimmune disease with skin manifestations, while sarcoidosis has been viewed as a predominantly pulmonary disorder with skin manifestations. Our results suggest that sarcoidosis may also be a systemic disease with immune dysregulation. Studies that stratify samples by therapy, organ involvement or disease progression are warranted. Future studies that utilize gene-regulatory networks and identify new genes may enhance our understanding of lupus or sarcoidosis as systemic disorders with implications for biomarkers or therapies.

## Data Availability Statement

Publicly available datasets were analyzed in this study. This data can be found here: <NCBI GEO GSE32887 and GSE52471>.

## Author Contributions

MN, KH, Y-SC, DP, and PF designed the experiments. MN, KH, and Y-SC performed the data analysis. MN wrote the manuscript. NS provided expertise related to sarcoidosis. MT provided expertise related to dermatological disorders. All authors reviewed and approved the manuscript.

## Conflict of Interest

The authors declare that the research was conducted in the absence of any commercial or financial relationships that could be construed as a potential conflict of interest.

## References

[1] Katta R. Cutaneous sarcoidosis: a dermatologic masquerader. Am Fam Physician. (2002) 65:1581–4. 11989634

[2] Tanner BMSSLS Discoid lupus erythematosus. StatPearls [Internet] (accessed July 3, 2020).

[3] Okon LG Werth VP. Cutaneous lupus erythematosus: diagnosis and treatment. Best Pract Res Clin Rheumatol. (2013) 27:391–404. 10.1016/j.berh.2013.07.008 24238695 PMC3927537

[4] Schleicher SM. Psoriasis: pathogenesis, assessment, and therapeutic update. Clin Podiatr Med Surg. (2016) 33:355–66. 10.1016/j.cpm.2016.02.004 27215156

[5] Rendon A Schäkel K. Psoriasis pathogenesis and treatment. Int J Mol Sci. (2019) 20:1475. 10.3390/ijms20061475 30909615 PMC6471628

[6] Ocampo DV Gladman D Psoriatic arthritis. F1000Res. (2019) 8:F1000 Faculty Rev-1665 10.12688/f1000research.19144.1

[7] Cai Y Fleming C Yan J. New insights of T cells in the pathogenesis of psoriasis. Cell Mol Immunol. (2012) 9:302–9. 10.1038/cmi.2012.15 22705915 PMC4132586

[8] Judson MA Marchell RM Mascelli M Piantone A Barnathan ES Petty KJ. Molecular profiling and gene expression analysis in cutaneous sarcoidosis: the role of interleukin-12, interleukin-23, and the T-helper 17 pathway. J Am Acad Dermatol. (2012) 66: e901–2. 10.1016/j.jaad.2011.06.017 21924794

[9] Jabbari A Suárez-Fariñas M Fuentes-Duculan J Gonzalez J Cueto I Franks AGJr. Dominant Th1 and minimal Th17 skewing in discoid lupus revealed by transcriptomic comparison with psoriasis. J Invest Dermatol. (2014) 134:87–95. 10.1038/jid.2013.269 23771123 PMC3858414

[10] Sehgal VN Pandhi D Khurana A. Biologics in dermatology: an integrated review. Indian J Dermatol. (2014) 59:425–41. 10.4103/0019-5154.139859 25284845 PMC4171908

[11] Sweiss NJ Baughman RP. Tumor necrosis factor inhibition in the treatment of refractory sarcoidosis: slaying the dragon? J Rheumatol. (2007) 34:2129–31. 17985412

[12] Dai C Shih S Ansari A Kwak Y Sami N. Biologic therapy in the treatment of cutaneous sarcoidosis: a literature review. Am J Clin Dermatol. (2019) 20:409–22. 10.1007/s40257-019-00428-8 30895525

[13] Chang AY Werth VP Treatment of cutaneous lupus. Curr Rheumatol Rep. (2011) 13:300–7. 10.1007/s11926-011-0180-z 21503694 PMC3245840

[14] Winkelmann RR Kim GK Del Rosso JQ. Treatment of cutaneous lupus erythematosus: review and assessment of treatment benefits based on oxford centre for evidence-based medicine criteria. J Clin Aesthet Dermatol. (2013) 6:27–38. 23320123 PMC3543290

[15] Kim WB Jerome D Yeung J. Diagnosis and management of psoriasis. Can Family Physician. (2017) 63:278–85. 28404701 PMC5389757

[16] Yavuz C. Biologics in dermatology: what does the future hold? Dermatol Ther. (2019) 32:e12932. 10.1111/dth.12932 30977240

[17] Ascoli C Huang Y Schott C Turturice BA Metwally A Perkins DL. A circulating micro-RNA signature serves as a diagnostic and prognostic indicator in sarcoidosis. Am J Respir Cell Mol Biol. (2017) 58:40–54. 10.1165/rcmb.2017-0207OC 28812922 PMC5941311

[18] Schott CA Ascoli C Huang Y Perkins DL Finn PW. Declining pulmonary function in interstitial lung disease linked to lymphocyte dysfunction. Am J Respir Crit Care Med. (2020) 201:610–3. 10.1164/rccm.201910-1909LE 31661301 PMC7047459

[19] Gerke AK Judson MA Cozier YC Culver DA Koth LL. Disease burden and variability in sarcoidosis. Annals Am Thoracic Soc. (2017) 14:S421–8. 10.1513/AnnalsATS.201707-564OT 29087725 PMC5802572

[20] Langfelder P Horvath S. WGCNA: an R package for weighted correlation network analysis. BMC Bioinform. (2008) 9:559. 10.1186/1471-2105-9-559 19114008 PMC2631488

[21] Miller J. Meta-Analyses of Data From Two (or More) Microarray Data Sets (2011). 25950827

[22] Liu J Jing L Tu X. Weighted gene co-expression network analysis identifies specific modules and hub genes related to coronary artery disease. BMC Cardiovascul Disord. (2016) 16:54–54. 10.1186/s12872-016-0217-3 26944061 PMC4779223

[23] Szklarczyk D Morris JH Cook H Kuhn M Wyder S Simonovic M. The STRING database in 2017: quality-controlled protein-protein association networks, made broadly accessible. Nucleic Acids Res. (2017) 45:D362–8. 10.1093/nar/gkw937 27924014 PMC5210637

[24] Shannon P Markiel A Ozier O Baliga NS Wang JT Ramage D. Cytoscape: a software environment for integrated models of biomolecular interaction networks. Genome Res. (2003) 13:2498–504. 10.1101/gr.1239303 14597658 PMC403769

[25] Xia WX Yu Q Li GH Liu YW Xiao FH Yang LQ. Identification of four hub genes associated with adrenocortical carcinoma progression by WGCNA. PeerJ. (2019) 7:e6555. 10.7717/peerj.6555 30886771 PMC6421058

[26] Phipson B Lee S Majewski IJ Alexander WS Smyth GK. Robust hyperparameter estimation protects against hypervariable genes and improves power to detect differential expression. Ann Appl Stat. (2016) 10:946–63. 10.1214/16-AOAS920 28367255 PMC5373812

[27] Amoura Z Combadiere C Faure S Parizot C Miyara M Raphaël D. Roles of CCR2 and CXCR3 in the T cell-mediated response occurring during lupus flares. Arthritis Rheum. (2003) 48:3487–96. 10.1002/art.11350 14673999

[28] Celhar T Magalhães R Fairhurst AM. TLR7 and TLR9 in SLE: when sensing self goes wrong. Immunol Res. (2012) 53:58–77. 10.1007/s12026-012-8270-1 22434514

[29] Celhar T Fairhurst A-M. Toll-like receptors in systemic lupus erythematosus: potential for personalized treatment. Front Pharmacol. (2014) 5:265. 10.3389/fphar.2014.00265 25538618 PMC4258990

[30] Li R Peng H Chen GM Feng CC Zhang YJ Wen PF. Association of FCGR2A-R/H131 polymorphism with susceptibility to systemic lupus erythematosus among Asian population: a meta-analysis of 20 studies. Arch Dermatol Res. (2014) 306:781–91. 10.1007/s00403-014-1483-5 24997134

[31] Genecards CD86 Gene [Online]. Weizmann Institutue of Science (accessed July 12, 2020).

[32] Nih. TNFRSF1B gene [Online] NIH U.S. National Library of Medicine (accessed July 12, 2020).

[33] Lis K Kuzawińska O Bałkowiec-Iskra E. Tumor necrosis factor inhibitors - state of knowledge. Arch Med Sci. (2014) 10:1175–85. 10.5114/aoms.2014.47827 25624856 PMC4296073

[34] Callejas-Rubio JL López-Pérez L Ortego-Centeno N. Tumor necrosis factor-alpha inhibitor treatment for sarcoidosis. Therapeut Clin Risk Manage. (2008) 4:1305–13. 10.2147/TCRM.S967 19337437 PMC2643111

[35] Almoallim H Al-Ghamdi Y Almaghrabi H Alyasi O. Anti-tumor necrosis factor-α induced systemic lupus erythematosus(). Open Rheumatol J. (2012) 6:315–9. 10.2174/1874312901206010315 23198006 PMC3504723

[36] Satterthwaite AB. Bruton's tyrosine kinase, a component of b cell signaling pathways, has multiple roles in the pathogenesis of lupus. Front Immunol. (2017) 8:1986. 10.3389/fimmu.2017.01986 29403475 PMC5786522

[37] Chalmers SA Wen J Doerner J Stock A Cuda CM Makinde HM. Highly selective inhibition of Bruton's tyrosine kinase attenuates skin and brain disease in murine lupus. Arthritis Res Ther. (2018) 20:10. 10.1186/s13075-017-1500-0 29370834 PMC5785891

[38] Kawasaki T Kawai T. Toll-like receptor signaling pathways. Front Immunol. (2014) 5:461. 10.3389/fimmu.2014.00461 25309543 PMC4174766

[39] Pinheiro RO Schmitz V Silva BJA Dias AA De Souza BJ De Mattos Barbosa MG Innate immune responses in leprosy. Front Immunol. (2018) 9:518 10.3389/fimmu.2018.00518 29643852 PMC5882777

[40] Rahman S Shering M Ogden NH Lindsay R Badawi A. Toll-like receptor cascade and gene polymorphism in host-pathogen interaction in Lyme disease. J Inflamm Res. (2016) 9:91–102. 10.2147/JIR.S104790 27330321 PMC4898433

[41] Margaritopoulos GA Antoniou KM Karagiannis K Samara KD Lasithiotaki I Vassalou E. Investigation of Toll-like receptors in the pathogenesis of fibrotic and granulomatous disorders: a bronchoalveolar lavage study. Fibrogenesis Tissue Repair. (2010) 3:20. 10.1186/1755-1536-3-20 20937083 PMC2964564

[42] Devarapu SK Anders HJ. Toll-like receptors in lupus nephritis. J Biomed Sci. (2018) 25:35. 10.1186/s12929-018-0436-2 29650017 PMC5898010

[43] Wu YW Tang W Zuo JP. Toll-like receptors: potential targets for lupus treatment. Acta Pharmacol Sin. (2015) 36:1395–407. 10.1038/aps.2015.91 26592511 PMC4816237

[44] Oltmanns U Schmidt B Hoernig S Witt C John M. Increased spontaneous interleukin-10 release from alveolar macrophages in active pulmonary sarcoidosis. Exp Lung Res. (2003) 29:315–28. 10.1080/01902140303786 12746045

[45] Godsell J Rudloff I Kandane-Rathnayake R Hoi A Nold MF Morand EF. Clinical associations of IL-10 and IL-37 in systemic lupus erythematosus. Sci Rep. (2016) 6:34604. 10.1038/srep34604 27708376 PMC5052569

[46] Nicod LP Isler P. Alveolar macrophages in sarcoidosis coexpress high levels of CD86 (B7.2), CD40, and CD30L. Am J Respir Cell Mol Biol. (1997) 17:91–6. 10.1165/ajrcmb.17.1.2781 9224214

[47] Petrek M Gibejova A Drabek J Mrazek F Kolek V Weigl E. CC chemokine receptor 5 (CCR5) mRNA expression in pulmonary sarcoidosis. Immunol Lett. (2002) 80:189–93. 10.1016/S0165-2478(01)00324-8 11803051

[48] Spagnolo P Renzoni EA Wells AU Copley SJ Desai SR Sato H. C-C chemokine receptor 5 gene variants in relation to lung disease in sarcoidosis. Am J Respir Crit Care Med. (2005) 172:721–8. 10.1164/rccm.200412-1707OC 15976369

